# Grammatical number processing and anticipatory eye movements are not tightly coordinated in English spoken language comprehension

**DOI:** 10.3389/fpsyg.2015.00590

**Published:** 2015-05-07

**Authors:** Brian Riordan, Melody Dye, Michael N. Jones

**Affiliations:** ^1^Aptima, Inc., FairbornOH, USA; ^2^Department of Psychological and Brain Sciences, Indiana UniversityBloomington, IN, USA

**Keywords:** grammatical number, eye movements, sentence comprehension, spoken word recognition, visual world paradigm

## Abstract

Recent studies of eye movements in world-situated language comprehension have demonstrated that rapid processing of morphosyntactic information – e.g., grammatical gender and number marking – can produce anticipatory eye movements to referents in the visual scene. We investigated how type of morphosyntactic information and the goals of language users in comprehension affected eye movements, focusing on the processing of grammatical number morphology in English-speaking adults. Participants’ eye movements were recorded as they listened to simple English declarative *(There are the lions.)* and interrogative *(Where are the lions?)* sentences. In Experiment 1, no differences were observed in speed to fixate target referents when grammatical number information was informative relative to when it was not. The same result was obtained in a speeded task (Experiment 2) and in a task using mixed sentence types (Experiment 3). We conclude that grammatical number processing in English and eye movements to potential referents are not tightly coordinated. These results suggest limits on the role of predictive eye movements in concurrent linguistic and scene processing. We discuss how these results can inform and constrain predictive approaches to language processing.

## Introduction

In the study of spoken language comprehension, the discovery that language processing is closely coordinated with patterns of eye movements represents a major advance for the discipline (Tanenhaus and Trueswell, 2006). Not only does the the visual context influence how the unfolding linguistic input is structured (Tanenhaus et al., 1995), but fixations to referents in the visual scene have been shown to reflect the fine-grained time course of spoken word recognition (e.g., Magnuson et al., 2007).

When processing linguistic and visual input simultaneously, listeners rapidly integrate across information streams, making anticipatory eye movements to likely referents. For example, Altmann and Kamide (1999) demonstrated that when listeners encounter verbs such as *eat*, they shift their visual attention to edible objects. Kamide et al. (2003) further demonstrated that listeners can integrate morphosyntactic and semantic information at the verb to drive eye movements to likely referents. Other work has demonstrated anticipatory looking behavior during thematic role assignment (Dahan and Tanenhaus, 2004; Knoeferle and Crocker, 2006).

These findings are consistent with a host of related experimental results suggesting that, like other aspects of human cognition, language comprehension and production are incremental, predictive processes. In making predictive inferences about upcoming speech or text, communicators draw on multiple sources of linguistic information, ranging over lexical, semantic, and discourse levels (for reviews, see Pickering and Garrod, 2007; Ramscar et al., 2010). This has been demonstrated empirically in a number of ways. For instance, in reading, more predictable items are processed faster and more efficiently (McDonald and Shillcock, 2003; Hare et al., 2009), and in speech production tasks, such items are uttered more quickly, often in a reduced form (Gahl et al., 2012), with fewer disfluencies (Arnold et al., 2007). Eye movement studies complement these traditional experimental domains, furnishing a rich picture of how various linguistic factors conspire to affect processing in real time (Huettig et al., 2011).

### Grammatical Gender

One important question that the visual world paradigm has begun to answer, is how syntactic agreement patterns assist comprehension processes. Agreement is thought to establish local and global coherence by linking temporally separated elements in discourse. However, precisely how it accomplishes this is an active area of research. A key line of enquiry concerns the influence of grammatical gender on lexical access. Gender systems are obligatory morphological systems found in many languages, which group nouns into a small number of mutually exclusive classes, and mark neighboring words – such as articles and adjectives – for agreement. In Romance languages, like French and Spanish, nouns are typically divided into two separate classes: masculine and feminine. Other major languages, such as Russian and German, add a third neuter category, and more are possible; Swahili has six (Corbett, 1991).

While historically gender has been viewed as an arbitrary or superfluous system (see Kilarski, 2007 for a review), there is an accumulating body of evidence to indicate otherwise. For one, while gender systems are not always semantically transparent, neither are they opaque to their speakers; there are typically multiple, converging linguistic cues to class membership (Frigo and McDonald, 1998). Further, gender systems may confer distinct advantages for native speakers. A leading hypothesis is that gender information reduces the lexical search space, delimiting the set of nouns to gender-consistent possibilities (but see Friederici and Jacobsen, 1999 for alternative proposals). On this view, speakers use gender to guide lexical access, helping them better predict upcoming nouns in discourse, as well as likely referents in the visual scene. This suggests that gender should both facilitate processing (when the marker is consistent with a following noun) and inhibit it (when the marker mismatches). Supporting evidence comes from a variety of sources, including lexical decision (Grosjean et al., 1994), naming times (Schriefers, 1993), word repetition (Bates et al., 1996), artificial grammar learning (Arnon and Ramscar, 2012), and ERP, where gender agreement violations have been found to produce neural error responses to the mismatch (Wicha et al., 2004; Van Berkum et al., 2005).

Yet perhaps the strongest support for the ‘limited search’ hypothesis comes from tasks that illuminate the time course of spoken language comprehension. In auditory gating paradigms, subjects hear short sequences in which a word fragment appears, and are asked to produce the target word. In a study of native French speakers, Grosjean et al. (1994) found that when gender information was provided, subjects correctly identified the target at shorter durations, and with greater confidence. More importantly, an inspection of subject errors revealed that gender information not only significantly reduced the number of misidentifications (both in terms of types and tokens), but also limited errors to gender-consistent candidates. Indeed, “in the presence of gender marking, no word candidate ever (had) the wrong gender” (Grosjean et al., 1994; p. 594). Similarly, in tip-of-the-tongue (TOT) states, Italian subjects can reliably guess the gender of the noun they are trying to retrieve, even when they cannot produce it (Vigliocco et al., 1997).

These findings are paralleled in studies of visual search. Dahan et al. (2000) investigated how gender-marked definite articles influenced the looking behavior of French-speaking participants. Subjects viewed a visual display with four possible referents, and heard instructions such as *Cliquez sur le bouton* (*Click on the_masc_ button*). When gender information was provided at the determiner, listeners rapidly shifted their attention to gender-consistent referents, ignoring potential phonological competitors. Lew-Williams and Fernald (2007) reported a comparable result for Spanish-speakers, finding that both children and adults are faster to orient to the correct referent on trials when nouns of different genders are displayed than on trials showing nouns of the same gender (see also Weber and Paris, 2004; van Heugten and Shi, 2009).

Taken together, these results support the conclusion that grammatical gender does not merely prime lexical candidates, but rather restricts the space of subsequent possibility. However, the studies reviewed here focus exclusively on several closely related Romance languages. There is also evidence to suggest that the function and strength of gender, as a morphosyntactic cue, may vary significantly by language (see, e.g., Miozzo and Caramazza, 1999). This is quite clearly the case when it comes to grammatical number.

### Grammatical Number

Grammatical number offers another promising domain of investigation for eye movement research. If gender is a widespread feature of the world’s languages, number is nearly universal. In the simplest number systems, a noun’s morphological form is modified to represent the numerosity of its referents, indicating whether the noun references a single entity or multiple entities, and neighboring words are marked for agreement (Corbett, 2000). In English, number is obligatory, and typically indicated by the presence or absence of a terminal sibilant +*s* (*cat*/*cats*), with several phonologically related families of irregulars (*mouse*/*mice*). A theoretical distinction is often drawn between *count nouns*, which alternate freely between singular and plural forms, and *mass nouns*, which are treated as a single, indivisible set, regardless of numerosity. Compare, for instance, the usage of the semantically related pairs *noodles*_count_/*pasta*_mass_, *colds*_count_/*flu*_mass_, and *jobs*_count_/*work*_mass_.

As with grammatical gender, number information may be a potentially useful resource for predicting upcoming referents. Listeners appear to process grammatical number information quickly and automatically. Grammatical number violations are registered particularly rapidly, a conclusion that has been established through reading times (Wagers et al., 2009) and ERP (Pulvermüller and Shtyrov, 2003; Barber and Carreiras, 2005). Complementary results have been reported in TOT paradigms, where English-speakers have been found to reliably discriminate the appropriate sentential contexts for count nouns, even on failure to retrieve them (Vigliocco et al., 1999). Collectively, these findings imply that available agreement information scaffolds prediction of upcoming items in discourse.

If this is the case, simply hearing the string *Look, there aresome*— might serve to restrict gaze to plural objects in a visual display. This is precisely what Kouider et al. (2006) found in a study of English-speaking children. On critical trials, toddlers saw pictures of novel objects on two screens; one picture depicted a single object and the other, multiple copies of the same object. Children heard sentences such as *Look, there are some blickets!* Beginning at 24 months, children were able to use the number marking on the copula and the indefinite article to launch anticipatory eye movements to the correct picture. Similar findings have been reported for French (Robertson et al., 2012). Complicating this picture, however, Johnson et al. (2005) report that in a picture selection task, English-speaking toddlers fail to use verb agreement marking as a cue to subject number (see Brandt-Kobele and Höhle, 2010 for a parallel finding in German).

Thus, despite some promising results, there is reason to suspect that grammatical number may not be as consistently informative about upcoming referents as grammatical gender. A variety of different theoretical accounts provide for different representations for gender and number (see discussion in Barber and Carreiras, 2005). One hypothesis is that whereas gender information is a property of the lexical item, stored in its lexical representation, number is is an independent morphological feature that combines with the stems of lexical items. These representational differences have processing consequences in models of lexical retrieval: gender information is retrieved with lexical access, while number information is involved only in a postlexical process of grammatical agreement as part of integration with the context. On this account, because grammatical number information does not directly activate lexical representations, processing of this information should only be weakly reflected in eye movements to referents in the visual scene.

Another source of difference may arise from number and gender’s very different relations to semantics (Eberhard et al., 2005). Speaking broadly, a noun’s number specification tends to be semantically motivated, reflecting the numerosity of the referent. By contrast, a noun’s gender specification tends to be semantically arbitrary, with little obvious correspondence between the conceptual properties of the referent and its noun class, and substantial cross-linguistic variation. Thus, whereas number tends to be an extrinsic, inflectional feature that is highly responsive to semantics, gender tends to be intrinsic and non-inflectional, with comparatively limited interaction with semantics (see Vigliocco et al., 2005). This suggests that as a predictive cue, number may be less informative in languages in which semantic factors strongly bias agreement patterns.

For this reason, it is important consider the distributional facts of the language under study: namely, English. In number agreement in English, the mapping between inflection and semantics is highly context-dependent, and is difficult to capture with simple, easily generalizable rules (Huddleston and Pullum, 2002). To grasp this point, it is helpful to consider just how far the language departs from a highly simplified case, in which agreement is computed solely as a function of a referent’s numerosity (singular/plural) and its semantic type (count/mass), and in which the semantic type distinction is clear-cut (e.g., mass nouns always refer to an undifferentiable whole).

The first complication is that, on inspection, there are certain systematic mismatches between syntax and semantics. For instance, mass nouns like *furniture* and *clothing* can be notionally plural while behaving like singulars (as when, e.g., there are multiple *pieces* of *furniture* or *articles* of *clothing* present), while pluralia tantum like *scissors* and *binoculars* can be notionally singular while behaving like plurals (as when there is a singular *pair* of *scissors* or *set* of *binoculars*). Nor is nominal inflection always a reliable guide to syntactic behavior, as evidenced by nouns whose meaning contravenes their marking, such as *news* (always singular), *police* (always plural), or *sheep* (which has the same singular and plural form).

Another wrinkle is that there is no straightforward way in which to tag nouns as countable, or not. While certain nouns fall on opposite ends of the *count*/*mass* spectrum, most nouns can behave in either way, depending on the semantic context (e.g., *I would like to buy a cake*/*I would like some more cake*). Further, countable nouns are not themselves a uniform class, and many show lexically specific preferences for (or restrictions on) the quantifiers they pair with. More broadly, item differences appear to be graded and distributional in kind, rather than rule-based and categorical (Baldwin and Bond, 2003). This suggests that agreement must be computed with reference to the entire noun phrase (NP), rather than simply the noun itself (Allan, 1980).

Finally, subject-verb agreement conventions are subject to variation both within and between speakers, and are closely influenced by semantics (Haskell and MacDonald, 2003; Eberhard et al., 2005). Singular collectives can take plural verbs (*the facultyare deliberating/neither of them are happy*) and plural quantities can take singular verbs (*ninety daysis a long time*). In addition to these ‘legal’ alternations, agreement errors are common; speakers are especially prone to interference when the main verb is proximate to a noun with a different number than its head noun, as in *The key to the cabinetswere missing* (Bock and Miller, 1991). In short, grammatical number in English is a highly complex system, in which agreement and marking conventions furnish, at best, an incomplete guide to the numerosity of the referent.

In the studies presented here, we sought to establish whether English-speaking adults make use of the partial information afforded by grammatical number to drive eye movements to likely referents, in contexts in which the predictive cue validity of number should be relatively weak. In online comprehension of both declarative and interrogative sentences, listeners first encountered grammatical number marking on the copula, in constructions such as *There are the cars* and *Where are the cars*? In addition, listeners heard sentences that incorporated multiple cues to number, such as *There aresome cars*, in which the indefinite article was also marked.

## Experiment 1

We recorded participants’ eye movements as they listened to declarative and interrogative sentences. Following Lew-Williams and Fernald (2007), participants were exposed to two types of trials. On *same-number* trials, participants saw two pictures that each had the same number of object exemplars. On these trials, participants could not determine the target referent until the onset of the noun. On *different-number* trials, the two pictures differed in the number of exemplars depicted. On these trials, participants could use grammatical number information that preceded the noun to quickly orient toward the correct referent. If grammatical number information is rapidly exploited in sentence comprehension, participants should be faster to fixate the picture that matches the linguistic input on different-number trials than on same-number trials.

### Method

#### Participants

Thirty native English speakers with normal or corrected-to normal vision participated for course credit.

#### Stimuli and Design

Noun targets were 16 object names with early age-of-acquisition. The words were divided into two sets of eight. Across participants, each set of eight words appeared in each condition. Within each set, no words shared the same initial phoneme. The noun targets were inserted in simple declarative and interrogative sentences. Sentences were of the form *There/Where [copula] [article] [noun].*

Two conditions varied the number of grammatical number cues in the sentences. In the definite determiner condition, both declarative and interrogative sentences included the definite determiner *the.* In this condition, the grammatical number information was only available on the copula. In the indefinite determiner condition, all sentences included an indefinite determiner, *a* or *some.* Here, grammatical number information was available on both the copula and the indefinite determiner.

There were 64 total test trials in each condition (see **Table 1**). Half of the trials were *same-number* trials, and half were *different-number* trials. In addition, half of the trials were sentences with singular number, and half with plural number. Within each condition, the target referent appeared equally often in the left and right locations. Each participant was exposed to half of the total stimuli in each condition (32 trials per condition), and eight filler trials. Thus participants saw a total of 80 trials during the experiment.

**Table 1:** Composition of test trials in Experiment 1.

Condition		Trial type		
		Same-number	Different-number	Example
Definite	Singular	16	16	*There is the lion.*
	Plural	16	16	*There are the lions.*
Indefinite	Singular	16	16	*There is a lion.*
	Plural	16	16	*There are some lions.*

Across the definite and indefinite conditions, each participant was exposed to half of the test items.

Sentences were recorded by a female speaker using a natural speech rate. All sentences employed the uncontracted form of the copula. Across sentences, the mean duration of copulas was 152 ms (range = 100–225), the mean duration of determiners was 151 ms (range = 50–275), and the mean duration of nouns was 591 ms (range = 300–800 ms).

**FIGURE 1:**
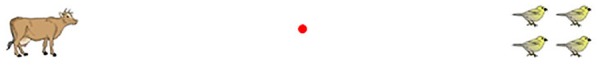
**Example visual display from a different-number trial**.

The visual stimuli were drawn from Rossion and Pourtois (2004). To form plural versions of each stimulus, four copies of each individual image were reduced in size and concatenated. The total surface area of the singular and plural images was identical. **Figure 1** depicts an example visual display for a different-number trial.

#### Procedure

Participants were instructed to click on the picture that was mentioned in the sentence (Weber and Paris, 2004). They were told to listen normally; no time constraints were imposed. As they listened, participants’ eye movements were recorded using a desktop-mounted SR Research EyeLink eyetracker sampling at 1000 Hz. Each trial began with the presentation of a fixation dot for 750 ms. There was 2000 ms preview time before sentence onset. Using the fixation dot as a cursor, participants clicked on the picture that matched the sentence. The trial ended with the mouse click. Each participant completed both the definite and indefinite conditions. Sentence order was randomized within condition, and the order of presentation of the conditions was counterbalanced across participants.

#### Analysis

The primary dependent variable was reaction time (RT) to initiate a saccade to the target referent (Lew-Williams and Fernald, 2007). We calculated RT as the latency of the first saccade or fixation that marked the start of an uninterrupted series of fixations on the target referent until the mouse click that ended the trial. RT was measured from copula onset.

Only trials that met the following conditions were included in the analysis. First, the participant must not have been fixating the target referent at the onset of the copula. Second, a saccade to or fixation on the target referent could not occur prior to 200 ms after the copula onset – approximately the earliest time a saccade could have been launched to the target referent after the copula onset (Altmann and Kamide, 2004). Third, RT must have occurred before 700 ms after the onset of the noun.

### Results and Discussion

**FIGURE 2:**
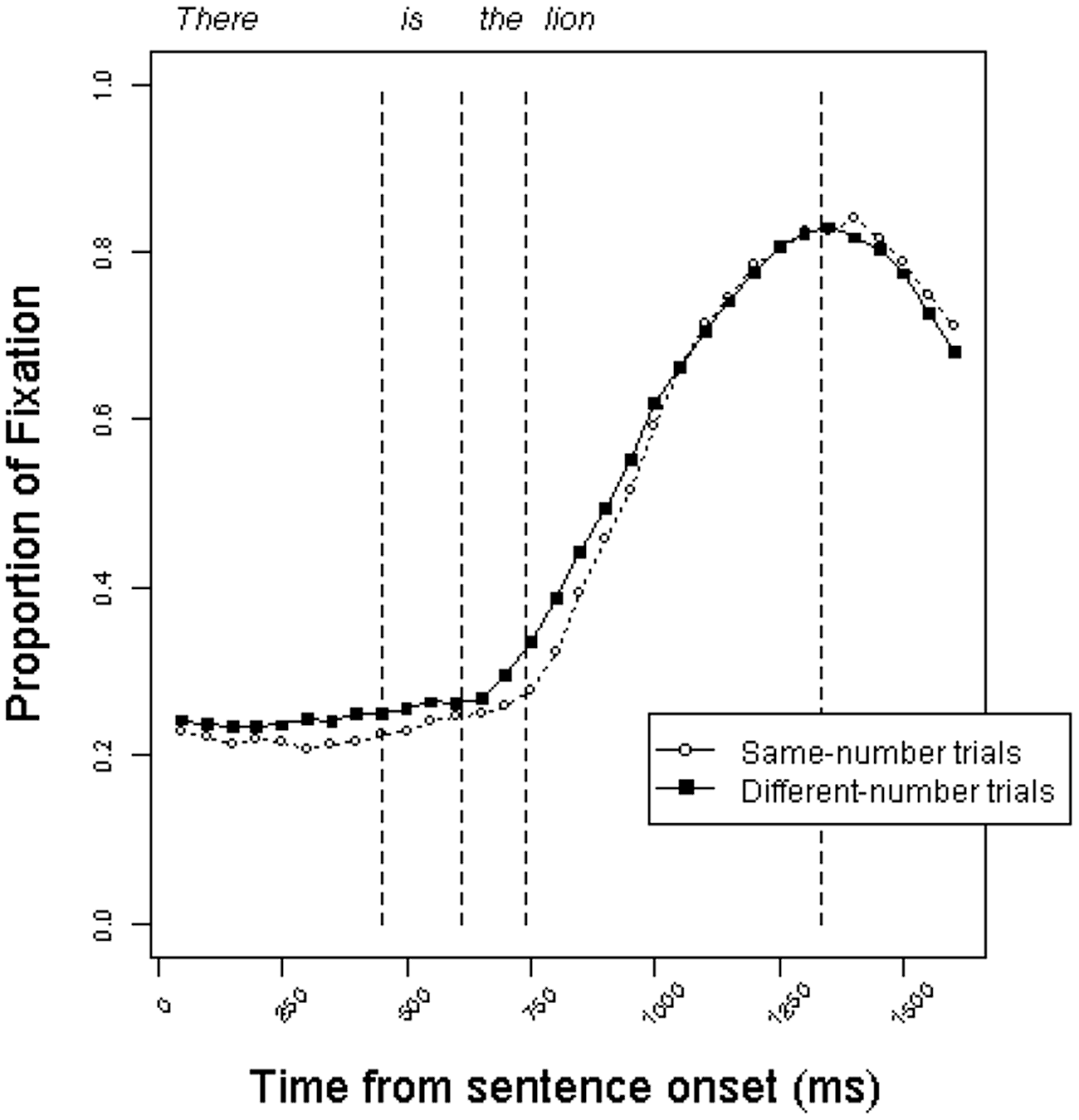
**Proportion of fixations to target objects in the definite condition on same-number versus different-number trials in Experiment 1.** Fixation proportions are averaged within 50 ms bins. Dashed lines represent average onsets of each word type within trial type (same vs. different).

**Figure 2** presents the time course of looking at each object in the display as the linguistic input unfolds in the definite condition. The curves represent the mean proportion of fixations to target objects on same-number trials versus different-number trials beginning with the start of the sentence. Participants shifted to the target object as the unfolding utterance allowed them to identify the correct picture. The trajectory of fixations is very similar across trial types, indicating that participants did not reliably use the grammatical number information encoded on the form of the copula to anticipate the target referent.

**FIGURE 3:**
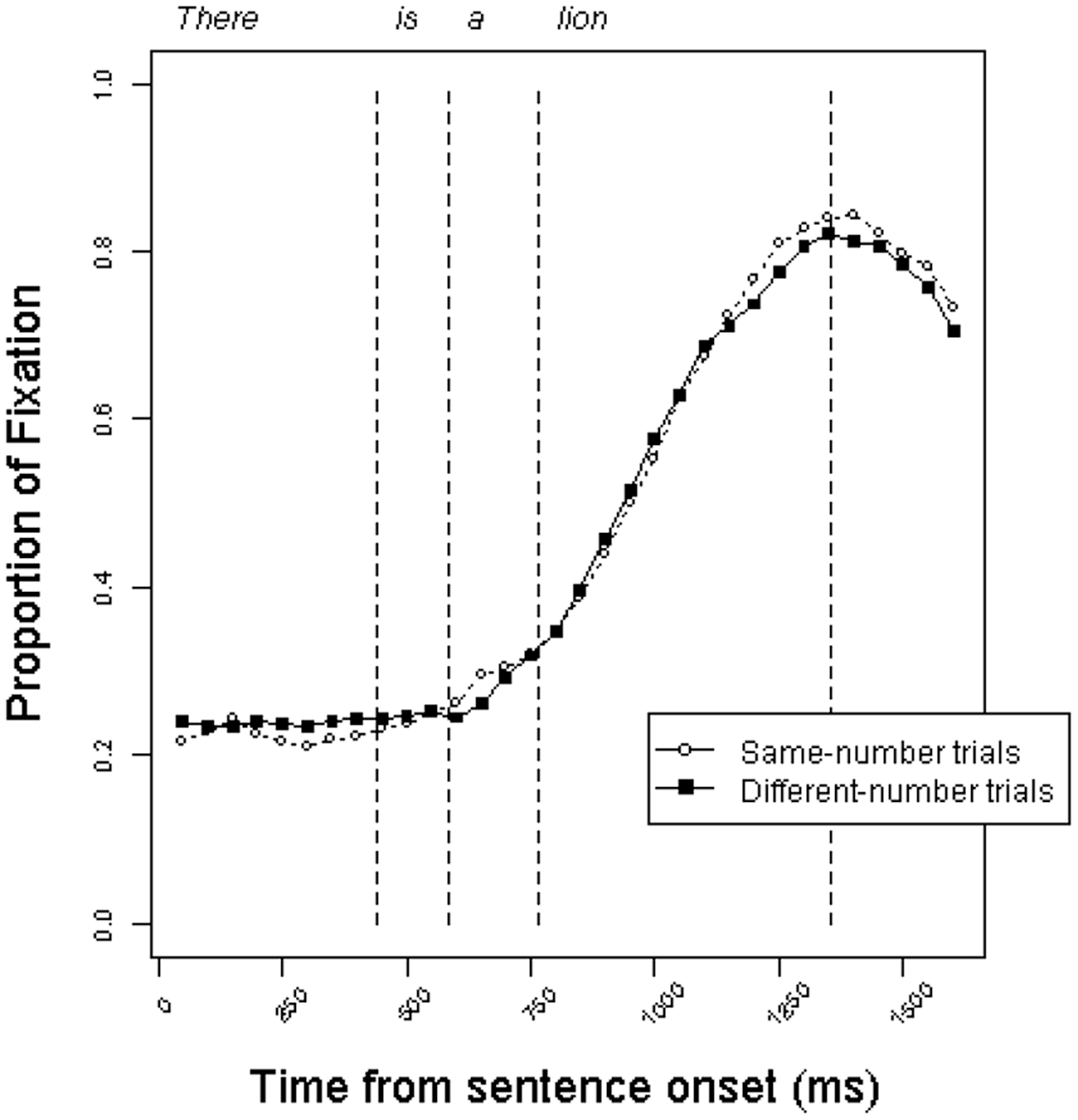
**Proportion of fixations to target objects in the *indefinite* condition on same-number vs. different-number trials in Experiment 1**.

**Figure 3** shows the time course of fixations for the two trial types in the indefinite condition. In this condition, too, the trajectory of fixations is similar across same-number and different-number trials. Participants did not make use of the two grammatical number cues preceding the noun – the copula and the indefinite article – to anticipate the correct referent.

These findings were confirmed with the RT analyses. Because sentence lengths varied with the type of copula (*is* vs. *are*) and the type of determiner (definite vs. indefinite, and within indefinite determiners, *a* vs. *some),* participants’ processing of the grammatical number information is likely to have varied across sentence types. Therefore, we report separate RT analyses by sentence type. Mean RT was calculated both by-subjects (*F*_1_) and by-items (*F*_2_). **Table 2** presents the results of within-subjects ANOVAs for each comparison. Although there were trends toward faster RT on different-number vs. same-number trials, in no case were these differences reliable in the expected direction.

**Table 2:** Experiment 1 reaction time (RT) analyses.

Condition		Same-number *M* (SD)	Different-number *M* (SD)	*F*_1_	*p*	*F*_2_	*p*
Definite	Singular	572 (109)	553 (142)	0.003	0.95	1.564	0.22
	Plural	574 (118)	555 (146)	0.132	0.72	0.003	0.96
Indefinite	Singular	524 (128)	572 (117)	0.778	0.38	8.836	0.006^1^
	Plural	613 (129)	560 (136)	0.017	0.90	0.400	0.53

^1^In this case, RT on different-number trials was *slower* than on same-number trials.

To explore the degree to which participants made anticipatory eye movements to the correct picture, we calculated the percentage of trials in which participants launched saccades to the target before they could process the noun (estimated as 200 ms after noun onset). Participants anticipated the target on only 35.1% of distracter-initial trials in the definite condition, and 39.6% of trials in the indefinite condition.

These results suggest that adults listening normally to simple declarative and interrogative sentences do not exploit grammatical number information to launch anticipatory eye movements to likely referents. We think it is unlikely that this null finding is due to a lack of power, given the consistent findings across both subjects and items, and the large number of exposures to each sentence type for each subject. Further, power analysis suggested sufficient observations for adequate sensitivity. However, it is possible that the surface structure led to strategic processing: anticipating that all sentences would have similar word order, participants may have adopted a strategy of simply waiting for the noun before shifting their gaze to the correct referent. Experiment 2 evaluated this possibility using the same stimuli and design as Experiment 1, but participants were instructed to select the correct referent as quickly as possible. Under these conditions, participants should use the grammatical number information on the copula and indefinite determiner to quickly orient to the correct picture.

## Experiment 2

### Method

#### Participants

Thirty native English speakers (not from Experiment 1) with normal or corrected-to-normal vision participated for course credit.

#### Stimuli and Design

Identical to Experiment 1.

#### Procedure

Participants were instructed to click on the picture that was mentioned in the sentence as quickly as possible without sacrificing accuracy. Otherwise, the procedure was identical to Experiment 1.

### Results

An ANOVA with Experiment as a between-subjects factor revealed that the change in instructions had a dramatic effect on RTs: Experiment 2 RTs (*M* = 496, SD = 121) were faster than Experiment 1 RTs (*M* = 566, SD = 129) [*F*_1_(1,454) = 35.9, *p* < 0.001; *F*_2_(1,252) = 40.8, *p* < 0.001]. The percentage of trials on which participants launched saccades to the target before they could process the noun also increased: 51.9% of trials in the definite condition and 49.9% of trials in the indefinite condition.

**FIGURE 4:**
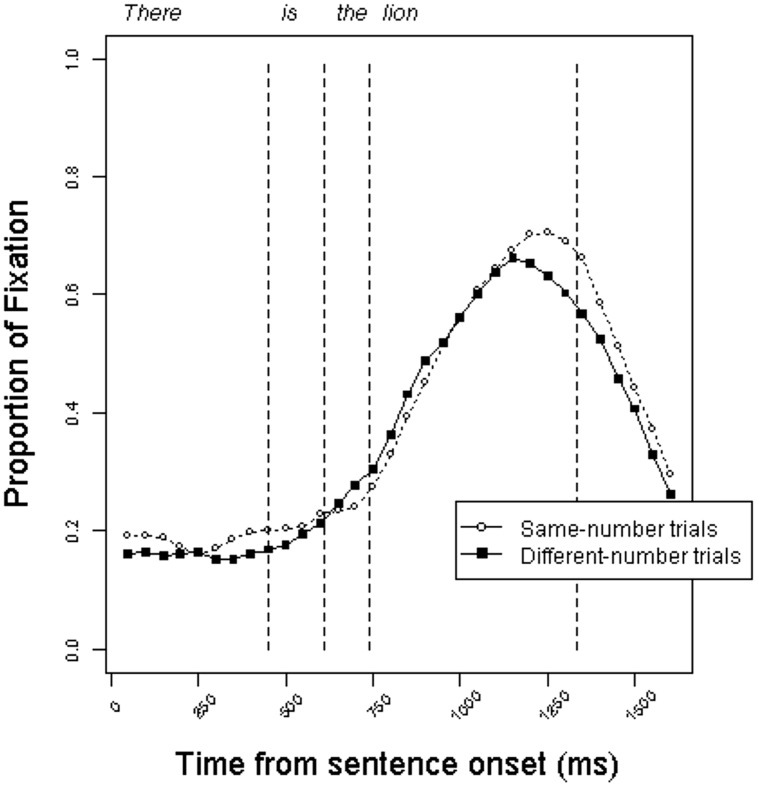
**Proportion of fixations to target objects in the definite condition on same-number vs. different-number trials in Experiment 2**.

**FIGURE 5:**
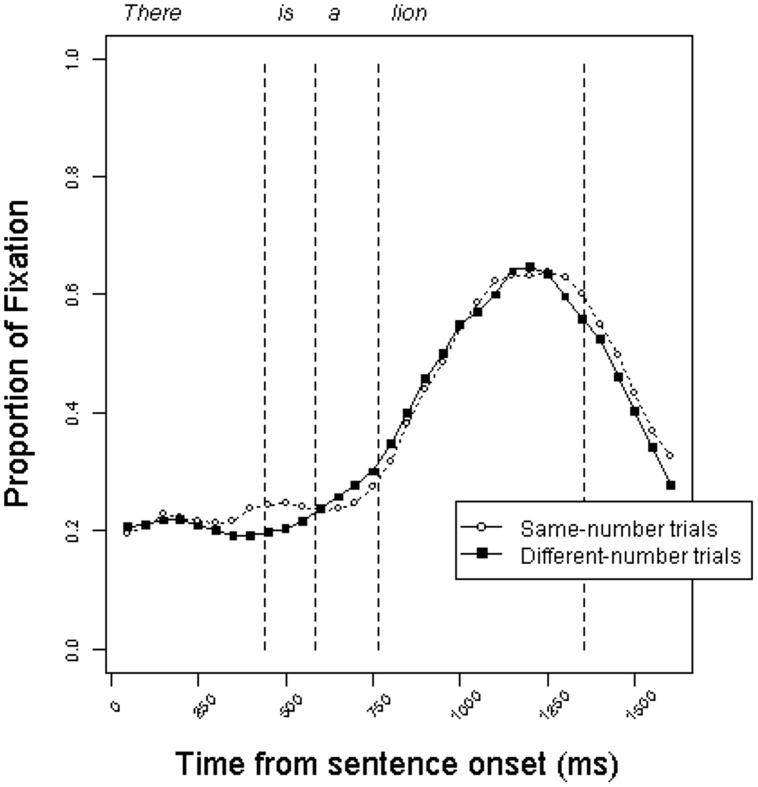
**Proportion of fixations to target objects in the *indefinite* condition on same-number vs. different-number trials in Experiment 2**.

**Figures 4** and **5** present the time course of mean fixation proportions to the target pictures in the definite and indefinite conditions, respectively. Surprisingly, the trajectory of fixation proportions is similar to those in Experiment 1. The curves do not give an indication of anticipatory eye movements on different-number trials relative to same-number trials.

The RT analyses are presented in **Table 3**. As in Experiment 1, although there was a trend toward faster processing in the different-number trials, this impression was not statistically reliable in any of the analyses. This was true for both the definite and indefinite conditions, despite the difference in grammatical number information that was available to participants. The results of Experiment 2 corroborate the results of Experiment 1, suggesting that the result of Experiment 1 was not an artifact of strategic processing.

**Table 3:** Experiment 2 RT analyses.

Condition		Same-number *M* (SD)	Different-number *M* (SD)	*F*_1_	*p*	*F*_2_	*p*
Definite	Singular	507 (119)	487 (119)	2.034	0.16	0.617	0.44
	Plural	492 (106)	453 (103)	1.297	0.26	0.134	0.72
Indefinite	Singular	503 (103)	491 (119)	0.728	0.40	1.637	0.21
	Plural	535 (136)	497 (155)	0.013	0.91	0.951	0.34

However, a potential concern still remains with Experiments 1 and 2. Since only declarative and interrogative sentences were used for the stimuli, it is possible that the results reflect strategies specific to the sentence types rather than a more general phenomenon of grammatical number processing in online language processing. Experiment 3 was designed to investigate this possibility using a similar design to Experiments 1 and 2 but with a wider range of sentence types.

## Experiment 3

### Method

#### Participants

Twenty native English speakers (not from Experiments 1 or 2) with normal or corrected-to-normal vision participated for course credit.

#### Stimuli and Design

Noun targets were 30 object names selected from McRae et al. (2005). These targets appeared in five conditions spanning auxiliary verbs in questions, declarative sentences, and demonstrative determiners. Each condition had singular and plural sentence versions, making 10 sentence sets. Three words were assigned to each sentence set and targets and distracter images were drawn from within the words in the sentence set. Distracters could not share the same initial phoneme as targets. Each target appeared in both same and different grammatical number conditions in separate trials of the experiment, yielding 60 unique grammatical number trials for each participant.

Because word types differ and length and word tokens differ in length with each utterance, across utterances, there is variation in the start and end of windows of interest. Therefore, it is common to align utterances based on the start of a window of interest for the purpose of analysis. An extension of this methodology to multiple windows of interest within an utterance involves resynchronizing at the start of each window (Altmann and Kamide, 1999). However, these techniques are only valid when the length of window, and word tokens within each window, are relatively homogeneous. Simply aligning utterances in this case runs the risk of glossing over utterance-specific eye movement behavior. Since our interest is in comparing the likelihood of launching a saccade based on information contained in function words, which are often phonetically reduced and of variable length, we chose to enforce an alignment of windows of interest across utterances by fixing the length of each window as shown in **Table 4**. Tokens shorter than the length of the window were followed by a short silence extending to the end of the window.

**Table 4:** Experiment 3 alignment of different sentence types.

Window onset (ms)	0	300	600	900	1500	1900	2300
	is	there	a	dog			
	are	there	some	dogs			
		does	the	dog	have	brown	fur
		do	the	dogs	have	brown	fur
	there	is	the	dog			
	there	are	the	dogs			
	there	is	a	dog			
	there	are	some	dogs			
			that	dog	is	black	
			those	dogs	are	black	

In addition to the grammatical number sentences, 60 new sentences were constructed using feature-target pairs selected from McRae et al. (2005) from 10 different feature types in order to compare anticipatory saccades as a function of feature type. However, these results will not be discussed in the current article. In order to ensure that participants did not develop an expectancy that target words would come later in the sentence, 60 filler sentences were created such that the first word was always the target referent. Target words for the filler sentences were the words from the feature experiment and filler sentences were generic sentences with plural subjects. The predicates of the filler sentences were features of the target word, but these features were different from the stimuli used in the feature experiment. Distracters could not share the same initial phoneme as targets. In 45 trials, both target and distracter images were plural. On the other 15 trials, the target image was singular while the distracter was plural. This was done to ensure that across the experiment participants did not develop expectations about the type of sentence they would hear based on the number-composition (i.e., target = singular, distracter = plural; etc.) of the image.

#### Procedure

Participants were required to make a saccade to an area of size 100 × 100 pixels surrounding a fixation dot in the center of the screen in order to initiate the sentence. This served to bring participants’ fixations to a uniform location before the start of the sentence. Once a saccade was registered to the center interest area, there was a 300 ms pause, then the sentence was played. Otherwise, the procedure was identical to Experiment 2.

### Results

The probability of initiating a saccade to the target object during a period starting 200 ms after the first word with grammatical number information and ending 150 ms after the onset of the target word was calculated for each participant by summing the number of trials in which a saccade to the target during this period occurred and dividing by the total number of trials. Since eye movements take approximately 180–200 ms to program, this is the critical period in which anticipatory eye movements could occur in response to the grammatical number information. Probabilities were calculated across all sentence types for each participant.

The anticipatory eye movement analyses are presented in **Table 5**. As in Experiments 1 and 2, no significant difference was observed between same-number and different- number trials, either for singular or plural sentences. The results of Experiment 3 further support the results of Experiments 1 and 2, suggesting that the results observed in these experiments were not due to the effect of strategic processing for different sentence types.

**Table 5:** Experiment 3 anticipatory eye movement analyses.

Condition	Same-number *M* (SD)	Different-number *M* (SD)	*F*	*p*
Singular	0.246 (0.217)	0.307 (0.218)	1.824	0.193
Plural	0.329 (0.229)	0.321 (0.248)	0.031	0.863

## General Discussion

Many studies have demonstrated the important role that prediction plays in language processing. Prediction has been central to the study of world situated language comprehension, with demonstrations of anticipatory eye movements in response to a variety of different kinds of linguistic information. However, the three experiments presented here failed to find evidence that eye movements are tightly coordinated with the processing of morphosyntactic information. Listeners did not respond reliably faster on trials where grammatical number cues were informative about the identity of the upcoming referent relative to trials where grammatical number cues were uninformative. This was true both under natural listening conditions (Experiment 1) and when emphasizing a speeded response (Experiment 2). In addition, listeners were no more likely to look at the upcoming referent when grammatical number cues were informative as compared to trials where grammatical number was uninformative, using a mixed variety of sentence types (Experiment 3).

Our adults participants, all native English-speakers, presumably had considerable previous experience with the distributional structure of their mother tongue, and could use that knowledge to anticipate discourse as it unfolded (Haskell et al., 2010). That they did not capitalize on number as a predictive cue, even under speeded conditions, suggests that number has low cue validity; though verb number was a reliable guide to conceptual number in our experiments, this is not true of the language at large. This dovetails nicely with theoretical work indicating that in sentence processing, English speakers pay relatively little attention to subject–verb agreement marking in establishing numerosity, instead relying on word order to resolve key dependencies (MacWhinney et al., 1984), and with a raft of findings indicating that cue validity is key to attentional orienting.

These results also complement that of Knoeferle and Crocker (2006), who found only a weak effect of tense and auxiliary words on eye movements. They found that auxiliary verbs such as *will* and *being* alone did not affect eye movements, but may have made the processing of the following verb and thematic role assignment faster. Knoeferle and Crocker (2006) concluded that there is generally a close coordination of scene processing and utterance comprehension, but this may be less so for words that only indirectly affect processing.

The finding that adult English-speakers do not reliably use grammatical number information to direct eye movements contrasts with the findings of Kouider et al. (2006) for young children (but see Johnson et al., 2005). As our experiments demonstrated, the nature of the task can have a large impact of the speed of eye movements in relation to linguistic input. Thus, the difference in findings could be attributed to differences in task, stimuli, or experimental procedure. A more interesting possibility is that novice and experienced English-language comprehenders differ qualitatively in their looking behavior during language comprehension.

Given the simplified nature of child-directed speech, adults may be more attuned to the range of possible continuations of the utterance following an opening such as *There is a*… For example, sentences with the singular copula *is* followed by the indefinite article *a* can be associated with plural referents, as when the referent is a collective noun, e.g., *There isagroup of ducks in the water*. Thus, more experience with language in a variety of communicative contexts, and specifically with more complex NPs, may reduce adults’ confidence in grammatical number morphology as a reliable cue to the identity of the upcoming referent. Indeed, because grammatical number information may not always be reliable, adults may make use of a form of “good-enough” processing (Ferreira et al., 2002) in these cases, computing an underspecified semantic expectation for possible referents (Sanford and Sturt, 2002).

This may be particularly true of certain constructions, such as the simple declaratives and interrogatives employed here, where grammatical number is only ever a partial guide to the numerosity of the referent. Naturally, there are many cases in which grammatical and conceptual number *do* align in such expressions, as was true of the sentences in our experiments. However, adults will also have been exposed to many instances in which grammatical number is highly unreliable as a predictive cue. For example, it will always be ambiguous for concrete mass nouns (*Where is the luggage she brought?*) and pluralia tantum (*There aresometongs on the counter*), where the number of the referent is left unspecified. Similarly, it will often be misleading when the verb is followed by a NP, and agreement is struck with the NP rather than the noun itself (*There isaherd of sheep*).

Varied conventions are not the only issue. A pair of large-scale corpus studies of British English confirms that agreement errors are quite common in declarative expressions, particularly in spoken language (Breivik and Martínez-Insua, 2008). Indeed, teenage speakers fail to achieve number agreement between the verb and post-verbal NP in more than a fifth of such utterances. The fact that number is not consistently informative in these contexts may help explain the growing tendency to omit number marking from them altogether (Meechan and Foley, 1994). In speech, English-speakers increasingly opt for the grammaticalized variants – *There’s* and *Where’s* – using these forms interchangeably with both singular and plural referents (*There’stwo ladies outside*).

It is not surprising then, that our participants did not rely on the number information encoded at the copula and determiner. Our null results argue against the notion that number in English is systematically informative about the numerosity of upcoming referents (see also Humphreys and Bock, 2005). More broadly, these results suggest that caution must be exercised in attempting to generalize the results of any one study – in any one language – to other studies in other languages, or to draw sweeping conclusions about the function of features like gender or number (MacWhinney et al., 1984). There is now an accumulating body of research attesting to cross-linguistic differences in morphosyntatic processing, showing systematic variation in number (Vigliocco et al., 1996; Berg, 1998) and gender processing (Miozzo and Caramazza, 1999; Schriefers and Teruel, 2000). Even within the same language, agreement processes may vary depending on the particulars of the construction (Kreiner et al., 2013), or the specific task demands (Brandt-Kobele and Höhle, 2010).

Cross-linguistic differences are to be expected. Languages vary widely in their “degree and specificity of morphological encoding” (Lupyan and Dale, 2010, p. 2), with some languages, like German, relying heavily on inflectional morphology to convey information, and others, like English, leaving more to the surrounding context—achieving lexically, what morphologically rich languages achieve through obligatory marking. In related work, Ramscar et al. (2015) have proposed that prenominal adjectives, in English, play a similar role to grammatical gender marking, in German. Both assist predictive processing; the difference is that one system is deterministic (only a certain set of nouns can legally follow the masculine article *der*), while the other is probabilistic (the distribution of nouns that follow *massive* and *moist* is markedly different, but not mutually exclusive). Thus, a possibility left open here is that rather than employing a rigid grammatical device, English simply relies on a more graded, semantically based means of specifying conceptual numerosity. This is consistent with the proposal that, in English, countability is a characteristic of NPs, rather than nouns (Allan, 1980), and that semantic principles selectively bias English agreement patterns (Berg, 1998).

In sum, English-speaking adults have difficulty consistently making use of grammatical number information to direct eye movements when processing simple declarative and interrogative sentences. This result indicates that the link between eye movements and linguistic processing is variable, depending especially on the linguistic information involved and the goals of language users.

## Conflict of Interest Statement

The authors declare that the research was conducted in the absence of any commercial or financial relationships that could be construed as a potential conflict of interest.
